# Ten simple rules for increased lab resilience

**DOI:** 10.1371/journal.pcbi.1008313

**Published:** 2020-11-19

**Authors:** Matthias C. Rillig, Milos Bielcik, V. Bala Chaudhary, Leonie Grünfeld, Stefanie Maaß, India Mansour, Masahiro Ryo, Stavros D. Veresoglou

**Affiliations:** 1 Freie Universität Berlin, Institut für Biologie, Berlin, Germany; 2 Berlin-Brandenburg Institute of Advanced Biodiversity Research (BBIB), Berlin, Germany; 3 Department of Environmental Science and Studies, DePaul University, Chicago, IL, United States of America; 4 Universität Potsdam, Institut für Biochemie und Biologie, Plant Ecology and Nature Conservation, Potsdam, Germany; Dassault Systemes BIOVIA, UNITED STATES

## Abstract

When running a lab we do not think about calamities, since they are rare events for which we cannot plan while we are busy with the day-to-day management and intellectual challenges of a research lab. No lab team can be prepared for something like a pandemic such as COVID-19, which has led to shuttered labs around the globe. But many other types of crises can also arise that labs may have to weather during their lifetime. What can researchers do to make a lab more resilient in the face of such exterior forces? What systems or behaviors could we adjust in ‘normal’ times that promote lab success, and increase the chances that the lab will stay on its trajectory? We offer 10 rules, based on our current experiences as a lab group adapting to crisis.

## Introduction

Crises that significantly impair lab research activities can strike without warning at any time. At the time of publication of this article, most research labs around the world have been shuttered due to the 2020 COVID-19 global pandemic. Other crises can also occur such as political instability or war, climate disasters (e.g. fires, floods, hurricanes), natural disasters (e.g. earthquakes), or the death of a lab member. In such serious situations that dramatically halt daily research activities, it may be useful to take stock of what aspects of lab culture or activities have contributed positively to dealing with this crisis. What could have been done better?

Certain aspects of how we can deal with unfolding crises are clearly outside our control. For example, how hard hit a certain area is, or if the cost of living and internet availability makes working from home feasible, or if the lab has core funding and permanent staff members. But certainly other aspects of how a lab is managed and how a team works can potentially provide greater resilience in times of crisis. Planning for a pandemic or a similar crisis event of course cannot and should not be a central goal of managing a lab, and no lab can be perfectly prepared. These events are rare, unpredictable, and in any case will require a great deal of improvisation and adjustments; nevertheless, we feel adhering to certain rules will be beneficial.

Resilience we take to mean the ability to retain the core lab mission and functioning when faced with a significant crisis. Adaptability is a part of resilience [1], meaning the capacity to adjust responses to changing external drivers and internal processes, and thus allowing the maintenance of a current trajectory, in this case of a lab group. Resilience is frequently enhanced by increasing the diversity within the group in ecological systems [2]. As we will see in the rules, diversity in a lab group can be defined and enhanced in many ways, thus increasing overall resilience.

**Fig 1:**
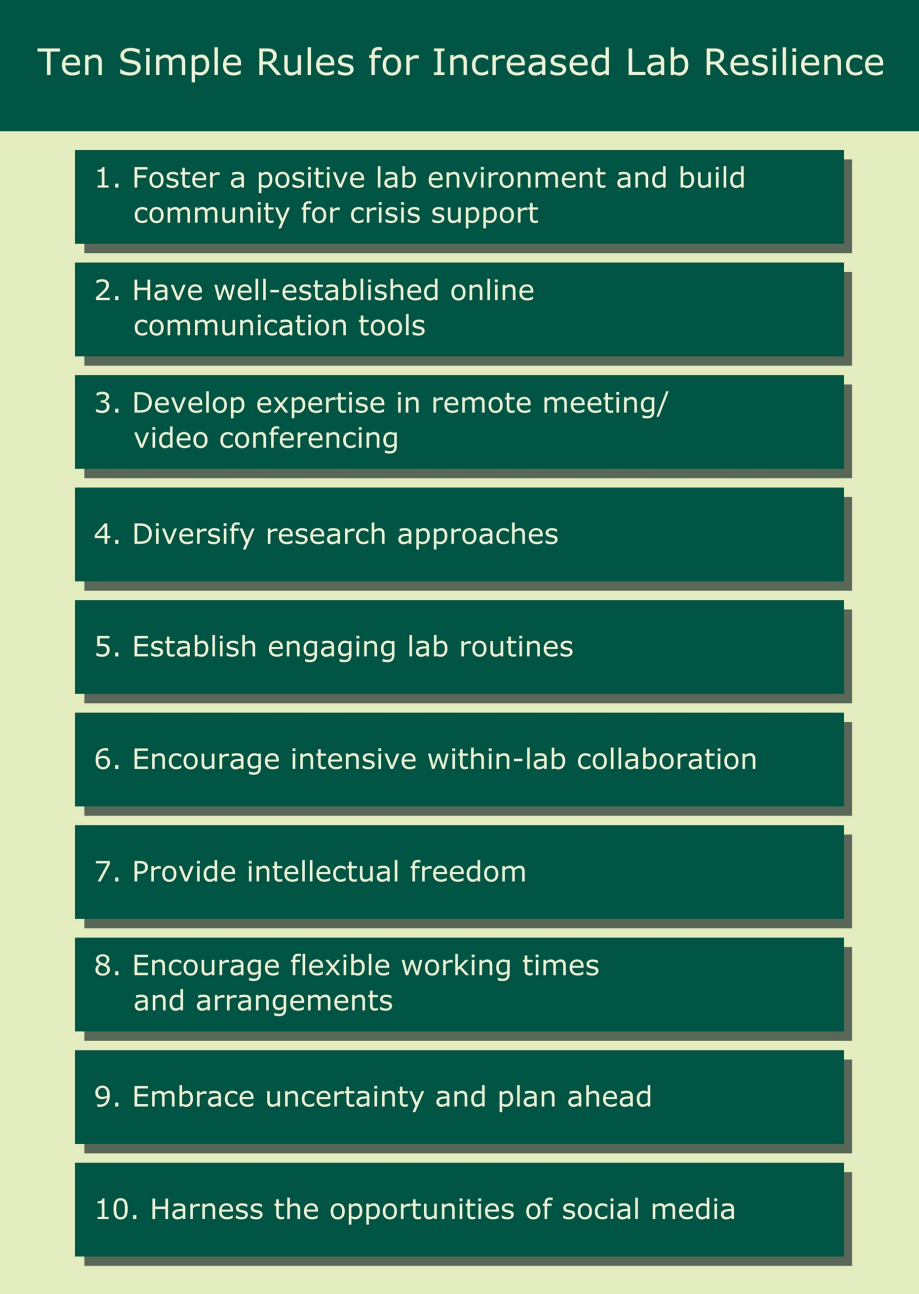
The ten simple rules presented in this paper.

In the following we provide a list of 10 rules (Fig 1) that we believe will allow a lab to respond more favorably to a catastrophic circumstance, taking our lab’s individual experience as a point of reference (for context: our lab works on ecology, is partly state-funded with some permanent staff, but mostly receives grant funding, is located in Berlin, Germany, and currently has about 50 persons from over 20 countries).

## Rule 1: Foster a positive lab environment and build community for crisis support

A good team spirit leads to an ‘emotional’ resilience through a sense of not being in this alone, and that people will help each other get through this situation. Recognize that lab members may be experiencing crisis while far from home, separated from family and friends, or alone. Some may have limited skills with the local language and therefore are less likely to consume relevant news. Identify the group as a place for support and trustworthy information. We created a special slack channel to share COVID19-related news, coping strategies, and ideas. Group members were also encouraged to regularly check in to make sure they had everything they needed to work safely at home. Transparent decision making and discussion of actions was also helpful. Benefits of a healthy lab culture have been discussed previously [3], and the importance of these points is likely amplified in times of severe crises.

## Rule 2: Have well-established online communication tools

Our lab moved to a group messaging tool a while ago; every lab member is registered there and all announcements and almost all lab communication and information exchanges are channeled through this tool. This meant that this was already available and part of everyday routine for everyone when lab shutdown loomed, and when the situation changed suddenly. Having established this mode of communication has proven invaluable, not only in terms of keeping communications going, but also in terms of sharing thoughts and feelings about the crisis. Also having other pieces of information online was advantageous, but not nearly everything was available in this fashion. Thus, one clear advice is: move key aspects of lab information flow online.

## Rule 3: Develop expertise in remote meeting/ video conferencing

Video meeting tools were not used routinely in our lab, except for the occasional skype calls with researchers from elsewhere. There was also no experience with online teaching. This turned out to be a significant issue early on, since this needed to be established and there was no prior experience. Video chats are vital for effortless communication, quick feedback and also more spontaneous exchanges. After setting this up, this turned out to be very positive: we use it for daily ‘coffee chats’, where people can spontaneously drop in without appointments, and also for lab meetings. Hopefully, once the current crisis is averted, these tools will remain part of the lab, so that we do not have this steep learning curve. Establish such tools and make them part of the daily routine; they can be useful for more frequent exchanges with lab alumni or other researchers in ‘normal’ times.

## Rule 4: Diversify research approaches

We are an empirically-focused lab with lab and greenhouse experiments and observational studies in the field taking center stage. However, the lab has fostered a culture of valuing synthesis, concept development, modeling, meta-analysis and data re-analysis. This turned out to be vital, because several lab members were affected relatively little in terms of ability to conduct their science, since their work did not depend on lab access. Additionally, other lab members could shift towards a project that took a more theoretical approach. This, in turn also meant that bachelor students could be shifted to such projects, since postdocs and doctoral candidates served as hubs for these types of approaches. In general terms, pursuing a wide range of different approaches in a lab will be beneficial in crisis times since it increases flexibility and the chances that some activities can continue because they are not affected. Merging empirical and synthesis approaches is thus key, and this approach also has other benefits [4].

## Rule 5: Establish engaging lab routines

The lab has several established routines that provide structure to interactions: we have Monday journal club and Thursday data club, as well as pub meetings on Thursdays every week and regular lunches. Subgroups also meet regularly for co-writing and topic-specific co-learning. On the one hand, losing these in-person meeting and social routines in a time of crisis exacerbates the immediate sense of loss, and this was certainly felt. On the other hand, they provide a structure that is to some degree transferable to online formats, and this happened to all these group meetings, even the pub evenings. While not exactly the same, the fact that they continue regularly provides some sense of continuity and normalcy.

## Rule 6: Encourage intensive within-lab collaboration

Within-group collaboration has been a central topic in the lab, and optimizing and actively seeking out such opportunities has been an important theme prior to the crisis. This means people are prepared to add to others’ projects and to help each other out with their respective expertise. This provides additional flexibility and opportunities in ‘normal’ times, but is especially valuable during a crisis. For example, some writing projects started right away, including the online-collaboration leading to this document.

## Rule 7: Provide intellectual freedom

Project deliverables have priority for researchers on outside funding (which means almost all), but beyond this lab members are encouraged to pursue side projects they find interesting, often but not always in collaboration with others. This generally likely increases productivity [5] and the diversity of ongoing projects. In a crisis, this means that there is simply a higher probability that some of these projects will be in the data analysis or write-up phase, where they can be more easily continued during lab closures.

## Rule 8: Encourage flexible working times and arrangements

Even though the lab emphasizes presence at regular lab meetings, beyond this people are flexible to find suitable arrangements for their work. This means that some lab members already had established home office routines and set-ups that they could use in a time of crisis, meaning they were somewhat less affected, adding to overall lab resilience.

## Rule 9: Embrace uncertainty and plan ahead

Risk mitigation is part of every-day lab routine: experiments can fail for various reasons, some outside the control of the person conducting them. It is thus good practice to plan for things that can go wrong in a number of ways; this can include complementing low-risk projects with more high-risk endeavors, planning experiments so that they can yield multiple useful answers, making sure data are being generated early on during a longer experiment, collaborating with others, combining approaches (see Rule 4), and many others. What is good practice anyway will pay double dividends when a catastrophic event occurs.

## Rule 10: Harness the opportunities of social media

The lab is active on twitter, and twitter has certainly been a source of inspiration and information during the unfolding of the crisis. It was reassuring to hear that people struggle with similar issues, and it was useful to be provided ideas for tools and approaches, and how other people handled certain issues (like teaching and video communication). Above all, social media provided a sense of community. At the same time, it is also important to not let oneself be overwhelmed by the torrent of information; thus: use social media as a support system, but be careful.

## Conclusions

Hopefully the next pandemic is very far off. Nevertheless, chances are that over the course of the lifespan of a lab group, maybe 30 years or so, adverse events do happen. None of the rules are in conflict with having a productive lab and a healthy lab group, and thus it seems like good advice to think about this set of 10 recommendations. Many labs will have already implemented quite a number of these aspects; to us they certainly took on a whole new meaning in the current situation, leading us to appreciate them in an entirely different way, and we hope this advice will be useful to others to dealing with this and any future calamities. Keeping a lab functioning under such stressful conditions is also beneficial for helping people in the lab through the crisis, not just for minimizing losses of productivity and funding.
